# Targeted inhibition of human hematological cancers *in vivo* by doxorubicin encapsulated in smart lipoic acid-crosslinked hyaluronic acid nanoparticles

**DOI:** 10.1080/10717544.2017.1384864

**Published:** 2017-09-28

**Authors:** Yinan Zhong, Fenghua Meng, Chao Deng, Xinliang Mao, Zhiyuan Zhong

**Affiliations:** a Biomedical Polymers Laboratory, and Jiangsu Key Laboratory of Advanced Functional Polymer Design and Application, College of Chemistry, Chemical Engineering and Materials Science, Soochow University, Suzhou, China;; b Department of Pharmacology, Jiangsu Key Laboratory of Translational Research and Therapy for Neuro-psycho-diseases, College of Pharmaceutical Sciences, Soochow University, Suzhou, Jiangsu, China;; c Jiangsu Key Laboratory of Preventive and Translational Medicine for Geriatric Diseases, Soochow University, Suzhou, Jiangsu, China

**Keywords:** Hematological cancer, targeted chemotherapy, CD44, reduction-sensitive, nanomedicine

## Abstract

The chemotherapy of hematological cancers is challenged by its poor selectivity that leads to low therapeutic efficacy and pronounced adverse effects. Here, we report that doxorubicin encapsulated in lipoic acid-crosslinked hyaluronic acid nanoparticles (LACHA-DOX) mediate highly efficacious and targeted inhibition of human hematological cancers including LP-1 human multiple myeloma (MM) and AML-2 human acute myeloid leukemia xenografted in nude mice. LACHA-DOX had a size of ca. 183 nm and a DOX loading content of ca. 12.0 *wt*.%. MTT and flow cytometry assays showed that LACHA-DOX possessed a high targetability and antitumor activity toward CD44 receptor overexpressing LP-1 human MM cells and AML-2 human acute myeloid leukemia cells. The *in vivo* and *ex vivo* images revealed that LACHA-DOX achieved a significantly enhanced accumulation in LP-1 and AML-2 tumor xenografts. Notably, LACHA-DOX effectively suppressed LP-1 as well as AML-2 tumor growth and drastically increased mice survival rate as compared to control groups receiving free DOX or PBS. Histological analyses exhibited that LACHA-DOX caused little damage to the major organs like liver and heart. This study provides a proof-of-concept that lipoic acid-crosslinked hyaluronic acid nanoparticulate drugs may offer a more safe and effective treatment modality for CD44 positive hematological malignancies.

## Introduction

Hematological malignancies that include leukemia, lymphoma, and myeloma account for about 10% of newly occurring cancer cases in the US (Siegel et al., 2016). Acute myeloid leukemia (AML) is the most common acute leukemia affecting adults while multiple myeloma (MM) is a cancer affecting mainly elderly people (Palumbo et al., 2011; Ferrara & Schiffer, 2013). Chemotherapy is an important treatment modality for hematological cancers (Burnett et al., 2011; Mahindra et al., 2012). Its therapeutic efficacy is, however, severely limited due to several intractable problems including poor cancer cell selectivity and high relapse rate. As a matter of fact, MM remains incurable to date (Chapman et al., 2011). In contrast to solid tumors that can be removed partly or in whole by surgery, hematological cancer cells are distributed throughout the blood pool and cannot be easily eliminated. The high relapse rate of hematological malignancy is considered related to existence of a rare population of cancer stem cells (Quere et al., 2011; Gao et al., 2016; Shen et al., 2016). Hence, to effectively treat hematological cancers, drugs should be potent not only toward cancer cells but also toward cancer stem cells.

In the past years, various nanomedicines have been designed and explored for the treatment of hematological malignancies (Bertrand et al., 2014; Visani et al., 2014; Tatar et al., 2016; Anchordoquy et al., 2017; van der Meel et al., 2017). Liposomes such as Doxil^®^ (a doxorubicin hydrochloride liposome system) and Marqibo^®^ (a vincristine sulfate liposome system) have received FDA approval for the treatment of MM and chromosome-negative acute lymphoblastic leukemia (ALL), respectively (Baz et al., 2006; Shah et al., 2016). Recently, liposomal formulation of cytarabine and daunorubicin (5/1), CPX-351, has shown to outperform standard cytarabine plus daunorubicin in treating refractory AML (Feldman et al., 2011; Lancet et al., 2014). Liposomes, though have improved the safety and pharmacokinetic properties of free drugs, are lack of cell selectivity. The installation of targeting ligands such as aptamers, antibodies, peptides, onto the surface of different nanoparticles including liposomes (Hazan-Halevy et al., 2016; Weinstein et al., 2016), poly(lactic-co-glycolic acid) (PLGA) nanoparticles (Babar et al., 2012), Pluronic P85 (Tatar et al., 2016), polydopamine nanospheres (Fan et al., 2016), and dextran nanoparticles (Hu et al., 2016), was shown to afford varying degrees of targetability *in vivo*. The transmembrane glycoprotein CD44 is one of the adhesion molecules present on hematological cancer cells and also identified as one of the cell surface antigens preferentially expressed on cancer stem cells in human hematological carcinomas (Misaghian et al., 2009; Zöller, 2011). Dick et al. reported that administration of an activating monoclonal antibody (H90) directed to CD44 markedly reduced leukemic repopulation in mice models transplanted with human AML (Jin et al., 2006). These findings point out that CD44 is a potentially attractive handle for achieving targeted treatment of hematological cancers.

Nanoparticles based on hyaluronic acid (HA) have received a wide attention for targeted cancer therapy as HA, a biocompatible and biodegradable natural polysaccharide (Toole, 2004; Liang et al., 2016), has an intrinsic binding affinity to CD44 that is overexpressed in many tumor cells (Cadete & Alonso, 2016; Dosio et al., 2016; Rao et al., 2016). Nanomedicines based on HA were shown to effectively target the different types of solid tumors including ovarian, breast, prostate, and lung tumors (Liu et al., 2012; Ganesh et al., 2013; Cohen et al., 2014; Li et al., 2014, 2015; Wang et al., 2015; Chen et al., 2016a; Wang & Jia, 2016; Yan et al., 2017). We recently reported that doxorubicin encapsulated in lipoic acid-crosslinked hyaluronic acid nanoparticles (LACHA-DOX) effectively suppressed CD44 overexpressing drug resistant human breast tumor xenografts in nude mice (Zhong et al., 2015). Interestingly, HA-coated magnetic nanoparticles were developed for selective detection and separation of CD44-overexpressed leukemia cells (Zhou & Xie, 2016). Peng et al. reported that HA-functionalized curcumin liposomes significantly delayed AML progression in mice as compared with free curcumin and the non-targeted controls (Sun et al., 2017). It thus appears that HA could also be employed to direct nanomedicines to CD44 overexpressed hematological tumor cells for treating hematological malignancy.

**Figure 1.**
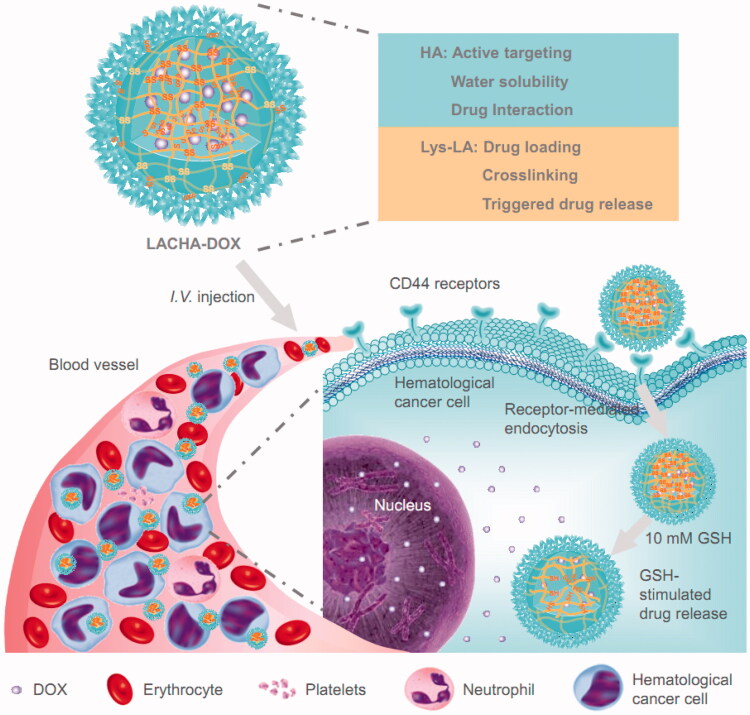
Illustration of DOX-encapsulated LACHA for the treatment of CD44 overexpressed hematologic malignancy. (i) LACHA-DOX can actively target to and be efficiently taken up by hematological tumor cells via a CD44-mediated endocytosis mechanism; and (ii) LACHA-DOX is automatically de-crosslinked inside the tumor cell, leading to fast intracellular drug release to the cytoplasm.

Here, we investigated the specificity and treatment efficacy of LACHA-DOX toward LP-1 human MM cells and AML-2 human acute myeloid leukemia cells *in vitro* and *in vivo* (Figure 1). Our results show that LACHA-DOX exhibits excellent targetability and potent growth inhibition of LP-1 and AML-2 tumor subcutaneously implanted in nude mice, leading to significantly improved survival rate and reduced systemic toxicity. Lipoic acid-crosslinked hyaluronic acid nanoparticulate drugs appear to offer a more safe and effective treatment for CD44 positive hematological malignancies.

## Materials and methods

### Materials

Sodium hyaluronic acid (HA, molecular weight: 35 kDa, Shandong Freda Biopharm Co., Ltd., China), 1,4-dithio-d,l-threitol (DTT, 99%, Merck), doxorubicin hydrochloride (DOX⋅HCl, >99%, Beijing ZhongShuo Pharmaceutical Technology Development Co., Ltd.), fluorescein isothiocyanate (FITC)-labeled CD44-antibody (Miltenyi Biotec, Germany), triethylamine, and dimethylsulfoxide (DMSO) were used as received. HA-Lys-LA (HA: 35 kDa, degree of substitution: 10%) was synthesized according to our previous report (Figure S1) (Zhong et al., 2015).

### Cell lines and cell cultures

LP-1 human MM cells were maintained in Iscove’s modified Dulbecco medium (IMDM, HyClone, Logan, UT). HL-60 human promyelocytic leukemia cells, THP-1 human acute monocytic leukemia cells, K562 human erythroleukemic cells, NB4 human promyelocytic cells, and AML-2 human acute myeloid leukemia cells were maintained in RPMI 1640 medium (HyClone). All media were supplemented with 10% fetal bovine serum (FBS, Gibco, Invitrogen, Carlsbad, CA), 100 μg/mL penicillin, and 100 U/mL streptomycin (HyClone).

### Measurement of CD44 expression levels of cells via flow cytometry

Hematological cancer cells including HL-60, THP-1, K562, NB4, AML-2, and LP-1 cells (5.0 × 10^5^ cells) were cultured in a 6-well plate under 5% CO_2_ at 37 °C. FITC-labeled CD44-antibody solution was added. The cells following incubation at 4 °C for 30 min were washed three times with PBS and collected by centrifugation (1000 *g*, 4 °C, 5 min). The cells following re-suspension in 0.5 mL of PBS were analyzed by BD FACSCalibur flow cytometer (Becton Dickinson, Franklin Lakes, NJ). The CD44 levels were expressed as the mean fluorescence intensity, calculated using the CellQuest software.

### Preparation of LACHA-DOX

DOX-encapsulated reversibly crosslinked HA nanomedicine (LACHA-DOX) was fabricated as our previous report (Zhong et al., 2015). Briefly, 4 mL of PB (10 mM, pH 7.4) was added dropwise to a stirred mixture of HA-Lys-LA (1.0 mL, 5 mg/mL) in formamide and DOX (250 μL, 5 mg/mL) in DMSO at r.t., followed by crosslinking with 10 mol.% DTT relative to the lipoyl units for another 24 h. LACHA-DOX was purified by extensive dialysis against PB (10 mM, pH 7.4) for 24 h (Spectra/Pore, MWCO 3500). The content of DOX was measured by fluorescence spectrophotometry (FLS920), and drug loading content (DLC) as well as drug loading efficiency (DLE) were calculated according to our previous report (Zhong et al., 2015).

### *In vitro* cytotoxicity

For *in vitro* studies, we have adopted human myeloma cells and leukemia cells that express high levels of CD44. The cells were suspended in 96-well plates at a density of 2 × 10^4^ cells/well using IMDM or RPMI 1640 media. After 24 h, the cells were exposed to HA nanocarriers for 48 h at 5% CO_2_ and 37 °C. MTT assays were performed as previously reported (Zhong et al., 2015).

The anti-proliferative activity of LACHA-DOX and free DOX to hematological cancer cells was also studied via MTT assays. Briefly, the cells were suspended in 96-well plates at a density of 2 × 10^4^ cells/well using IMDM or RPMI 1640 media. After 24 h, the cells were exposed to LACHA-DOX or free DOX at different DOX concentrations for 4 h at 5% CO_2_ and 37 °C. The cell media were replaced by fresh media and the cells were cultured for another 44 h before MTT assays. To verify receptor-mediated endocytosis, cells were pretreated with free HA (5 mg/mL) for 4 h before adding LACHA-DOX.

### Flow cytometry

Flow cytometry was employed to investigate the cellular uptake and intracellular drug release behaviors of LACHA-DOX. The cells were seeded in a 6-well plate (1 × 10^6^ cells/well) using IMDM or RPMI 1640 media for 12 h. LACHA-DOX or free DOX was added (10.0 μg DOX/mL) and the cells were cultured for 2 or 4 h at 5% CO_2_ and 37 °C. The cells following twice washing with PBS and re-dispersing in 500 μL PBS were analyzed via flow cytometer. The cells pretreated with free HA (5 mg/mL) were used as a control.

### *In vivo and ex vivo* imaging

The mice were managed in compliance with the guidelines of Soochow University Laboratory Animal Center and the Animal Care and Use Committee of Soochow University. Near-infrared fluorescent molecule DIR was encapsulated into HA-Lys-LA nanocarriers (LACHA-DIR) for *in vivo* imaging. To develop human myeloma or leukemia xenografted mice models, 2 × 10^7^ human myeloma or leukemia cells in 100 μL serum-free IMDM or RPMI 1640 media were injected into the hind flank of each mouse. At tumor volume of about 150 mm^3^, LACHA-DIR was administrated via the tail vein. At different time intervals (1, 4, 8, 12, and 24 h) post injection, mice were subjected to the Maestro *in vivo* fluorescence imaging system (CRi Inc.).

To trace the organ distributions of LACHA-DOX, *ex vivo* fluorescence imaging was performed. At 8 h after intravenous administration of LACHA-DOX or free DOX at a dose of 10 mg DOX equiv./kg, human myeloma or leukemia tumor-bearing mice of each group were sacrificed. Then, the tumor block and the major organs including heart, liver, spleen, lung, and kidney were excised, washed, dried, and observed using the Maestro *in vivo* fluorescence imaging system (CRi Inc.).

### *In vivo* antitumor efficacy

Human myeloma or leukemia tumor-bearing mice with tumor volume of about 80 mm^3^ were treated with LACHA-DOX and free DOX at a dose of 7.5 mg DOX equiv./kg. The drugs were intravenously injected on indicated days (day 0, 3, 6, 9, and 12). PBS was used as a blank control. The tumor volume (V) and body weight (W) were recorded every 3 days and the volume was calculated by the formula V = 0.5 × length × width^2^. Mice were regarded to be dead once the tumor volume reached 1000 mm^3^ or died during treatment.

### Histological analysis

On day 24, one mouse of each group was sacrificed. The tumor, liver, and heart were excised, fixed with paraformaldehyde for 48 h, embedded in paraffin, and cut into 5-micron thickness with a microtome. The tissue slices were stained by hematoxylin and eosin (H&E) and observed by a digital microscope (Leica QWin).

## Results and discussion

### Selectivity of LACHA-DOX to hematological cancer cells

Our previous work has demonstrated that HA nanomedicines possess extraordinary binding affinity toward CD44 positive solid tumors including breast and lung tumors (Chen et al., 2016b; Zhong et al., 2016). It is known that the treatment of hematological cancers is deeply perplexed by low selectivity and high relapse rate. Given the fact that several hematological cancer cells and cancer stem cells are overexpressing CD44, we expect that LACHA-DOX may also selectively target to hematological cancers.

**Figure 2.**
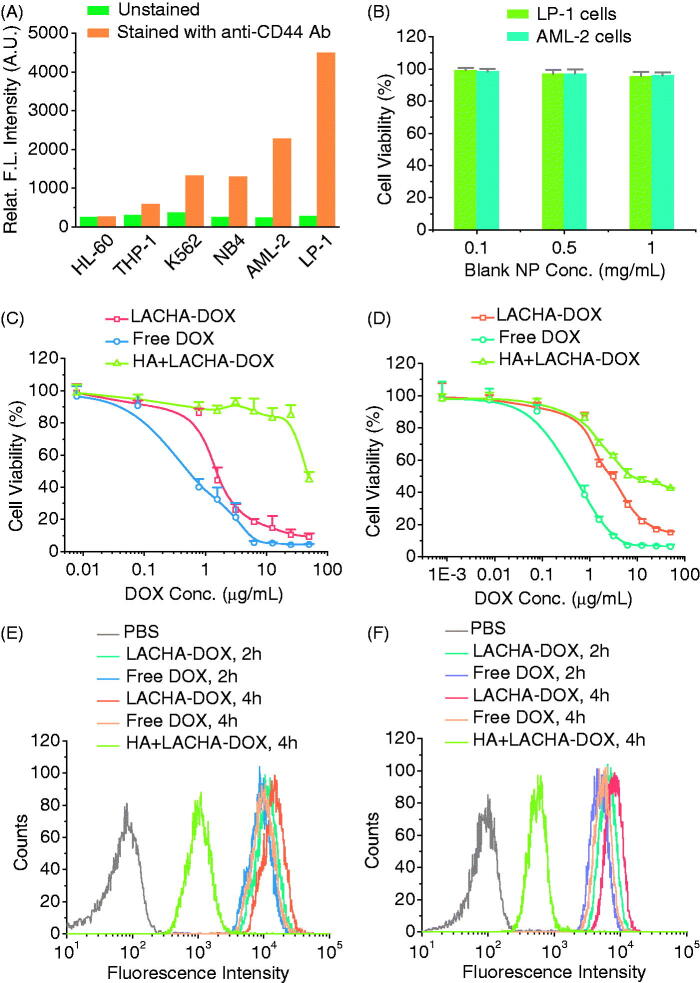
(A) CD44 expression in various blood cancer cells. Human MM cell line (LP-1) and human leukemia cell lines (HL-60, THP-1, K562, NB4, and AML-2) were treated with FITC-labeled antibody against CD44 receptors to determine the receptor levels on the surface of the cells by flow cytometry. (B) Cytotoxicity of bare LACHA nanoparticles in LP-1 and AML-2 cells following 48 h incubation (*n* = 4). The *in vitro* proliferative inhibition activity of LACHA-DOX toward LP-1 cells (C) and AML-2 cells (D). The cells were treated with LACHA-DOX for 4 h and then cultured in fresh medium for another 44 h. Free DOX was used as a control. The inhibition experiments were performed by pre-treating cells for 4 h with 5 mg/mL free HA prior to incubation with LACHA-DOX (*n* = 4). Flow cytometry of LP-1 cells (E) and AML-2 cells (F) following 2 and 4 h incubation with LACHA-DOX (10 μg DOX/mL). Free DOX was used as a control. The competitive inhibition experiments were performed by pre-treating cells with free HA (5.0 mg/mL) for 4 h before adding LACHA-DOX.

We firstly examined the CD44 expression in six different hematological cancer cells including HL-60, THP-1, K562, NB4, AML-2, and LP-1 using FITC-labeled CD44 antibody. Flow cytometry showed that AML-2 and in particular LP-1 cells presented a high CD44 expression, K562 and NB4 cells a moderate CD44 expression while HL-60 and THP-1 cells a low CD44 expression (Figure 2(A)). In the following, we have chosen LP-1 and AML-2 cells for both *in vitro* and *in vivo* experiments. LACHA-DOX was fabricated as reported previously with a decent DOX loading content of 12.0 *wt.*%, a hydrodynamic size of ca. 183 nm, and a negative surface charge of −20.1 mV (Table S1) (Zhong et al., 2015). TEM micrograph of LACHA showed a slightly decreased size of ca. 150 nm (Figure S2). The high DOX loading of LACHA-DOX is likely contributed to existence of both hydrophobic and charge interactions between DOX and LACHA. Similar strategies were also exploited to enhance drug loading into polymersome and micelle systems (Wu et al., 2013; Chen et al., 2015). The disulfide crosslinking would not only largely increase the stability of LACHA-DOX in circulation but also efficiently release the payload within tumor cells. The *in vitro* drug release revealed that ca. 86.5% DOX was released in 22 h from LACHA-DOX in the presence of 10 mM GSH, which mimics the intracellular environment (Zhong et al., 2015). In comparison, most reported targeted nanomedicines showed a gradual drug release behavior. For example, ca. 25.0% and 48.0% drug was released in 24 h under physiological conditions from CD19-targeted DOX-loaded PEG-PCL micelles (Krishnan et al., 2015) and HA-modified curcumin liposomes (Sun et al., 2017), respectively.

MTT assays revealed that blank LACHA nanocarriers were nontoxic toward both LP-1 and AML-2 cells at a nanoparticle concentration of 1 mg/mL (Figure 2(B)). In contrast, LACHA-DOX exhibited a high and dose-dependent cytotoxicity with a low half-maximal inhibitory concentration (IC_50_) of ca. 1.45 and 2.76 μg DOX equiv./mL toward LP-1 and AML-2 cells, respectively (Figure 2(C,D)). The relatively higher antitumor activity of LACHA-DOX toward LP-1 cells may stem from their higher CD44 expression than AML-2 cells. LACHA-DOX presented lower cytotoxicities toward both LP-1 and AML-2 cells that had been pre-incubated with free HA (Figure 2(C,D)), signifying that LACHA-DOX is internalized by LP-1 and AML-2 cells via a receptor-mediated mechanism.

We have also employed flow cytometry to study the cellular uptake and intracellular drug release of LACHA-DOX in both LP-1 and AML-2 cells. Interestingly, the results showed that the fluorescence intensity of both LP-1 and AML-2 cells following 2 or 4 h incubation with LACHA-DOX was higher than those incubated with free DOX under otherwise the same conditions (Figure 2(E,F)), confirming fast uptake and drug release of LACHA-DOX in both cells. However, pre-treating LP-1 and AML-2 cells with free HA prior to incubation with LACHA-DOX resulted in drastically reduced cellular DOX level, further confirming that LACHA-DOX is taken up by LP-1 and AML-2 cells via a CD44-mediated mechanism. The enhanced cytotoxicity and DOX fluorescence intensity in both LP-1 and AML-2 cells treated by LACHA-DOX as compared to control groups corroborated a high specificity of LACHA-DOX toward LP-1 and AML-2 cells *in vitro*.

### *In vivo* biodistribution of LACHA-DOX in hematological cancer-bearing nude mice

**Figure 3.**
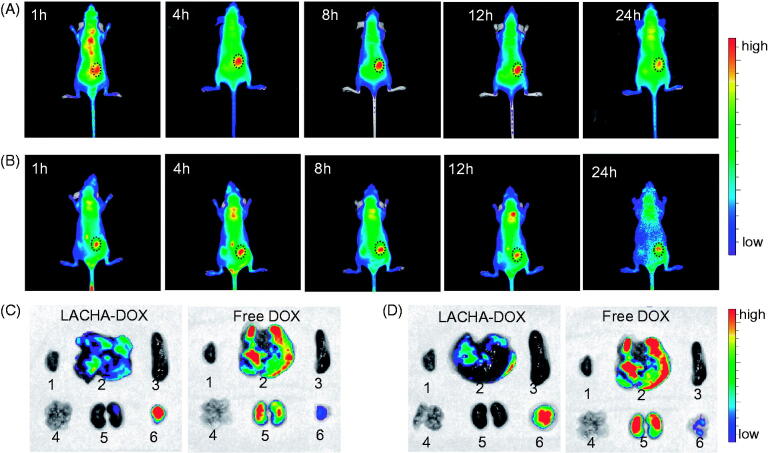
*In vivo* fluorescence images of nude mice bearing LP-1 human MM (A) and AML-2 human acute myeloid leukemia (B) at different time points following injection of LACHA-DIR. The mouse autofluorescence was removed by spectral unmixing using the Maestro software. *Ex vivo* DOX fluorescence images of tumor and major organs (1: heart, 2: liver, 3: spleen, 4: lung, 5: kidney, and 6: tumor) from nude mice bearing LP-1 human MM (C) and AML-2 human acute myeloid leukemia (D) at 8 h post injection of LACHA-DOX or free DOX.

To evaluate the tumor-targetability of LACHA-DOX *in vivo*, we have established subcutaneous LP-1 human MM and AML-2 acute myeloid leukemia xenografts in nude mice. DIR (a near infrared dye)-loaded HA nanoparticles (LACHA-DIR) were injected intravenously to tumor bearing mice and monitored using a Maestro EX *in vivo* fluorescence imaging system (CRi, Inc.). Notably, strong DIR fluorescence was observed in both tumors at 1 h post injection (Figure 3(A,B)). The tumor fluorescence reached the maximum at 8 h post injection (Figure 3(A,B)), demonstrating high tumor accumulation and retention of LACHA-DIR in both LP-1 and AML-2 tumors.

To further study their biodistribution, we performed the *ex vivo* DOX fluorescence imaging in the tumor and major organs of LP-1 and AML-2 tumor-bearing nude mice excised at 8 h post *i.v.* injection of LACHA-DOX. Remarkably, LP-1 tumor-bearing mice treated with LACHA-DOX revealed significantly stronger DOX fluorescence in the tumor than in the healthy organs such as liver, spleen, kidney, lung, and heart (Figure 3(C)). In contrast, free DOX-treated mice exhibited negligible DOX fluorescence in the tumor while strong DOX fluorescence in the liver and spleen (Figure 3(C)). Similar DOX distribution was also observed for LACHA-DOX in AML-2 tumor models (Figure 3(D)). These results indicated that LACHA-DOX has a high selectivity and accumulation in both LP-1 and AML-2 tumors *in vivo*, and can quickly release DOX into the tumors as the fluorescence of DOX loaded within the nanoparticles is self-quenched (Zhu et al., 2016). The *in vivo* and *ex vivo* fluorescence imaging concludes that LACHA-DOX has superior targetability to LP-1 human MM and AML-2 acute myeloid leukemia, which should drastically decrease the side effects in patients with MM or AML.

### *In vivo* therapeutic efficacy of LACHA-DOX in LP-1 human MM bearing nude mice

**Figure 4.**
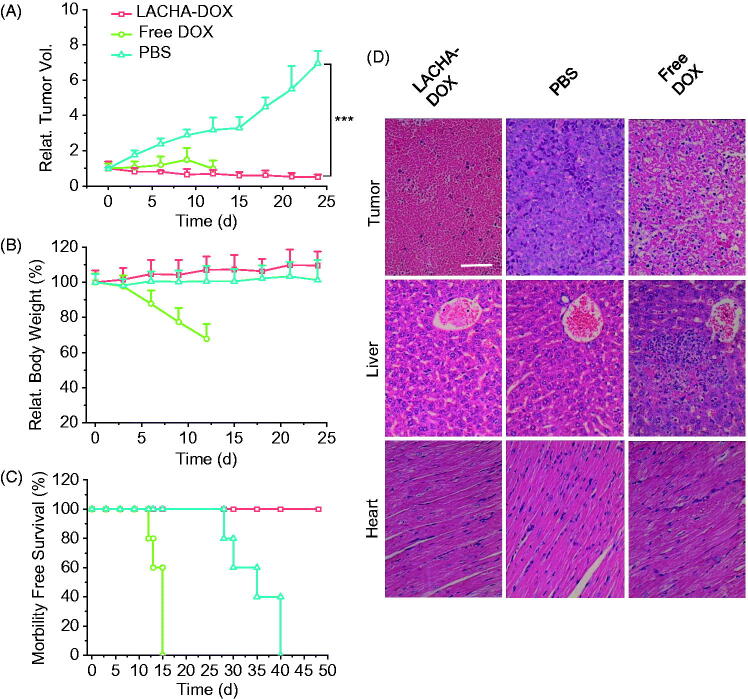
*In vivo* antitumor effects of LACHA-DOX in LP-1 human MM-bearing nude mice. Inhibition of tumor growth (A), body weight changes (B), and survival rates (C) of mice treated with LACHA-DOX, free DOX and PBS, respectively. The drugs were administrated on day 0, 3, 6, 9, and 12 (dosage: 7.5 mg DOX equiv./kg) (*n* = 6). ***p < .001 (Student’s *t* test); (D) H&E-staining of tumor, liver, and heart sections excised from LP-1 human MM-bearing mice on day 24 after different treatments. The scale bar represents 50 μm.

We have evaluated the therapeutic performance of LACHA-DOX using LP-1 human MM tumor-bearing nude mice. The mice were treated with LACHA-DOX or free DOX (7.5 mg DOX equiv./kg) when tumors grew up to about 80 mm^3^ in volume. The treatment was repeated every three days. Mice received PBS were used as a control. Remarkably, LACHA-DOX effectively inhibited tumor growth with approximately 71% reduction of tumor size and two mice exhibited complete suppression (Figure 4(A)), mainly attributing to the extraordinarily high CD44 expression on the surface of LP-1 cells. Free DOX at the same dosage though could also suppress tumor growth caused significant body weight decline (Figure 4(A,B)), likely due to severe systematic toxicity. In contrast, mice treated with LACHA-DOX grew well (Figure 4(B)), indicating LACHA-DOX brings about little side effects. The survival curves showed that LP-1 tumor-bearing mice treated with LACHA-DOX all survived within an experimental period of 48 d while free DOX and PBS group had median survival times of 15 and 35 d, respectively (Figure (4C)). Hence, LACHA-DOX can effectively suppress LP-1 tumor growth and drastically increase mice survival rate.

We further studied the histology of tumor and several healthy organs of mice following different treatments using H&E staining. The results showed that LACHA-DOX induced much more necrosis in the tumor of mice than free DOX (Figure 4(D)). It should further be noted that hardly any histopathological changes were detected in liver and heart of mice treated with LACHA-DOX, whereas seriously impaired liver tissues and disarray of cardiac muscle cells were observed in free DOX group (Figure 4(D)). These results point out that LACHA-DOX has not only brought about improved therapeutic efficacy of LP-1 human MM but also greatly reduced drug-associated systemic toxicity.

### *In vivo* therapeutic efficacy of LACHA-DOX in AML-2 human acute myeloid leukemia bearing nude mice

**Figure 5.**
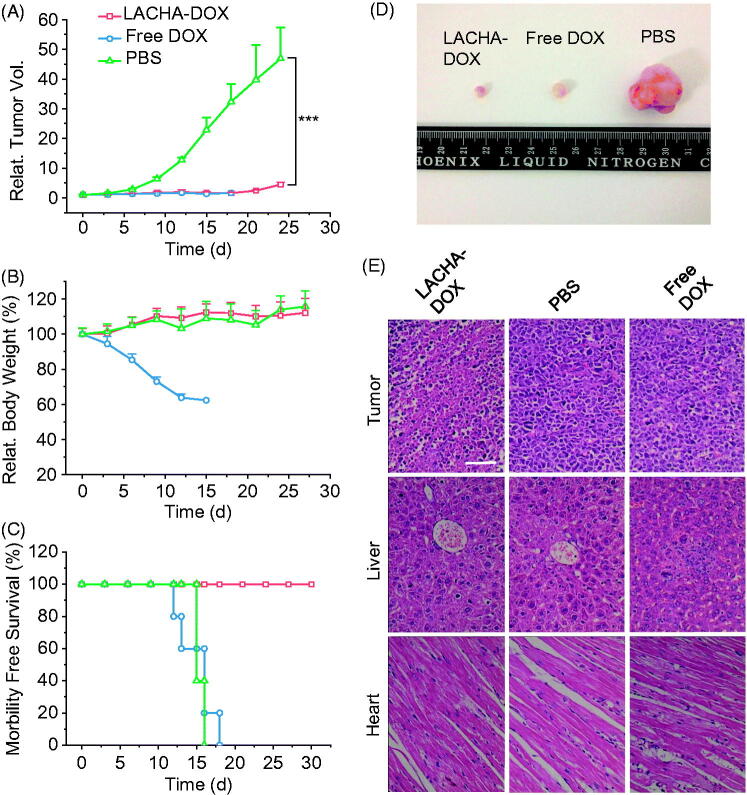
*In vivo* antitumor effects of LACHA-DOX in AML-2 human acute myeloid leukemia-bearing nude mice. Inhibition of tumor growth (A), body weight changes (B), and survival rates (C) of mice treated with LACHA-DOX, free DOX and PBS, respectively. The drugs were administrated on day 0, 3, 6, 9, 12 and 15 (dosage: 7.5 mg DOX equiv./kg) (*n* = 6). ***p < .001 (Student’s *t* test); Photos of typical tumor blocks (D), and H&E-staining of tumor, liver, and heart sections (E) isolated from different treatment groups on day 15. The scale bar represents 50 μm.

To further validate the treatment effects of LACHA-DOX to hematological cancers, we also studied its therapeutic efficacy in AML-2 acute myeloid leukemia tumor-bearing nude mice. The results again showed that LACHA-DOX led to the most effective suppression of tumor growth among all three groups (Figure 5(A)). Moreover, in sharp contrast to drastic body weight loss for free DOX group, mice treated with LACHA-DOX had little body weight change (Figure 5(B)). The survival curves showed that mice treated with LACHA-DOX all survived within an experimental period of 30 d (Figure 5(C)). In comparison, free DOX and PBS groups both had a short median survival time of ca. 15 d. The death of mice treated with free DOX is likely due to its high systematic toxicity rather than tumor itself. The effective tumor growth inhibition of LACHA-DOX was further confirmed by the small size of tumor isolated on day 15 (Figure 5(D)). The histological analyses using H&E staining revealed that LACHA-DOX caused extensive necrosis in the tumor tissue with little appreciable adverse effect to the liver and heart (Figure 5(E)). In contrast, less tumor necrosis but significantly more liver and heart damage was observed for mice treated with free DOX (Figure 5(E)). These results confirm that LACHA-DOX can effectively treat CD44 positive hematological cancers with little side effects, which is consistent with high selectivity to CD44 positive tumors of LACHA from *in vivo* and *ex vivo* fluorescence imaging.

The *in vivo* studies showed that LACHA-DOX brought about superb targetability and potent growth inhibition in both LP-1 human MM and AML-2 human acute myeloid leukemia xenografts, leading to significantly improved survival time and reduced systemic toxicity. The high potency of LACHA-DOX in CD44-overexpressed hematological cancers probably results from its high stability, prolonged blood circulation, evident targetability, receptor-mediated cellular uptake, and fast intracellular drug release. In the following, we will investigate the therapeutic efficacy of LACHA-DOX in the patient-derived xenograft model of leukemia. Interestingly, Farokhzad et al. reported that bone-targeting stealth bortezomib nanomedicines had similar tumor inhibition and slightly improved survival time in an orthotopic MM MM1S mice model as compared to free bortezomib (Swami et al., 2014). LACHA-DOX with easy fabrication and high antitumor effect has a great potential for targeted treatment of CD44 positive blood cancers.

## Conclusions

We have demonstrated that doxorubicin encapsulated in LACHA-DOX mediate highly efficacious and targeted treatment of human hematological cancers including LP-1 human MM and AML-2 human acute myeloid leukemia in nude mice. LACHA-DOX has several exceptional features such as high stability, high tolerability, fast glutathione-responsive drug release, and superior selectivity toward CD44 that is overexpressed in several human hematological cancer cells as well as cancer stem cells. It is remarkable that LACHA-DOX achieves effective inhibition of CD44 positive LP-1 and AML-2 tumors without causing side effects, leading to significantly improved survival rate for both LP-1 and AML-2 tumor-bearing mice. LACHA-DOX has appeared as a highly appealing platform for targeted treatment of CD44 positive hematological cancers.

## Supplementary Material

IDRD_Zhong_et_al_Supplemental_Content.pdf
