# Supplementary material for: Targeted inhibition of human hematological cancers in vivo by doxorubicin encapsulated in smart lipoic acid-crosslinked hyaluronic acid nanoparticles
Source: Drug Deliv. 2017 Sep 28;24(1):1482–90. doi: 10.1080/10717544.2017.1384864 (PMC8240992; doi:10.1080/10717544.2017.1384864)
Supplement: IDRD_Zhong_et_al_Supplemental_Content.pdf [file IDRD_A_1384864_SM3011.pdf]

Supporting Information

Targeted Inhibition of Human Hematological Cancers *In Vivo* by Doxorubicin Encapsulated in Smart Lipoic Acid-Crosslinked Hyaluronic Acid Nanoparticles

Yinan Zhong[a], Fenghua Meng [a,*], Chao Deng [a], Xinliang Mao [b,c,*], and Zhiyuan Zhong [a,*]

[a] *Biomedical Polymers Laboratory, and Jiangsu Key Laboratory of Advanced Functional Polymer Design and Application, College of Chemistry, Chemical Engineering and Materials Science, Soochow University, Suzhou, 215123, P. R. China.*

[b] *Jiangsu Key Laboratory of Translational Research and Therapy for Neuro-psycho-diseases, Department of Pharmacology, College of Pharmaceutical Sciences, Soochow University, Suzhou, Jiangsu 215123, China*

[c] *Jiangsu Key Laboratory of Preventive and Translational Medicine for Geriatric Diseases, Soochow University, Suzhou, Jiangsu 215123, China*

* Corresponding authors: Tel/fax: +86-512-6588 0098. fhmeng@suda.edu.cn (F. Meng); xinliangmao@suda.edu.cn (X. Mao); zyzhong@suda.edu.cn (Z. Zhong).

Characterization

Transmission electron microscopy (TEM) was performed using a Tecnai G220 TEM operated at an accelerating voltage of 200 kV. The samples were prepared by dropping 10 μL of 0.2 mg/mL nanoparticle suspension on the copper grid followed by staining with phosphotungstic acid (1 *wt*. %).

Statistical analysis

Data were expressed as mean ± SD. For studies on relative tumor volume changes, differences between groups were assessed using the paired, two-sided Student's t-test. $^{*}p < 0.05$ was considered significant, and $^{**}p < 0.01$, $^{***}p < 0.001$ were considered highly significant.

Table S1. Characteristics of LACHA-DOX.

Polymer	Size (nm)[a]	PDI[a]	Zeta (mV)[b]	DLC (*wt*.%)		DLE
				Theory	Determined[c]	(%)[c]
HA-Lys-LA	183	0.15	-20.1	20.0	12.0	54.5

[a] Determined by DLS analysis at 25 ºC in PB (pH 7.4, 10 mM);

[b] Determined by zeta potential measurements at 25 ºC in PB (pH 7.4, 10 mM);

[c] Determined by fluorescence measurement.

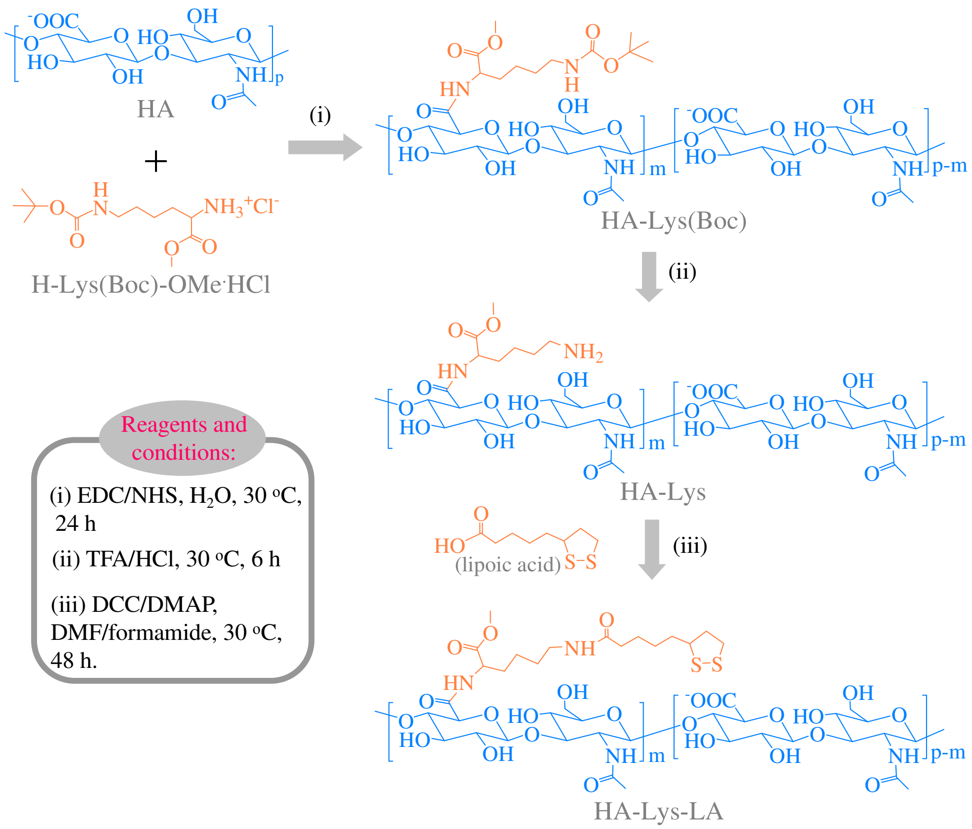

Figure S1. Synthesis of HA-Lys-LA conjugate.

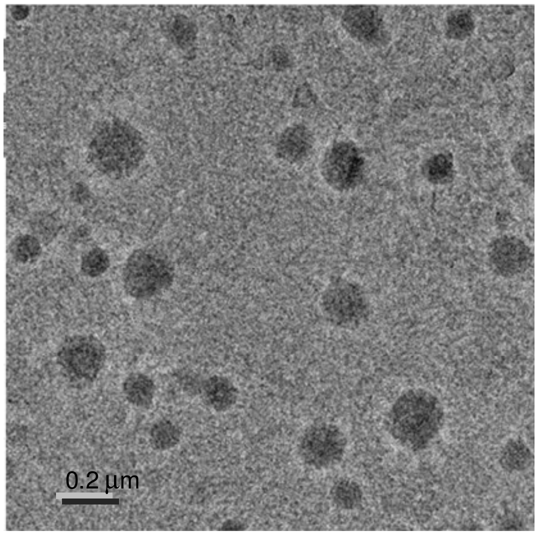

Figure S2. TEM micrograph of LACHA.
